# Dynamic Gait Stability Estimated Using One or Two Inertial Measurement Units Worn on the Human Body

**DOI:** 10.3390/s26041211

**Published:** 2026-02-12

**Authors:** Haoyun Peng, Shogo Okamoto, Hiroki Watanabe, Yasuhiro Akiyama

**Affiliations:** 1 Department of Computer Science, Tokyo Metropolitan University, 6-6 Asahigaoka, Hino, Tokyo 191-0065, Japan; 2 Department of Mechanics and Robotics, Shinshu University, 3-15-1 Tokita, Ueda, Nagano 386-8567, Japan; akiyama_yasuhiro@shinshu-u.ac.jp

**Keywords:** margin of stability, principal motion analysis, motion synergy

## Abstract

The margin of stability (MoS) is a metric used to assess dynamic postural stability during walking. Although MoS is typically computed from optical motion capture data, previous studies have shown that MoS can be approximated from six-axis kinematic signals—linear acceleration and angular velocity—measured by inertial measurement units (IMUs). With IMU-equipped devices such as smartphones and smartwatches becoming widespread, it is increasingly common for individuals to carry two or more such devices in daily life. This study aimed to identify combinations of two body locations that most effectively predict MoS. IMU sensors were attached to ten body locations while participants walked on a treadmill. Principal motion analysis, a reductive regression method for multidimensional time-series data, was employed for MoS prediction, and cross-validation was used for reliable model evaluation. Appropriate combinations of two IMU sensors achieved mean errors of approximately 30 mm and 11 mm in anterior and mediolateral MoS, respectively, compared with reference values derived from optical motion capture. These errors were comparable to the intrinsic standard deviations of MoS, suggesting that IMU-based MoS estimation is sufficiently accurate for the classification of individuals at high fall risk.

## 1. Introduction

Walking requires continuous regulation of dynamic stability. For older adults, gait instability is a major health concern, as falls can result in serious injuries such as bone fractures, leading to long-term hospitalization or the need for nursing care [1,2,3,4,5,6,7]. For example, epidemiological studies in Japan report that approximately 20–30% of older adults experience at least one fall annually, underscoring the public health relevance of this issue [1].

Although falls are less frequent and typically less severe in younger populations, previous studies have reported non-negligible fall incidence even among younger adults [8,9]. These findings indicate that fall risk can manifest across a wide age range, even among individuals without apparent mobility limitations.

Developing reliable methods to quantify dynamic postural stability during walking is therefore essential, primarily for finding the elderly at high risk of falls and for identifying subtle balance deficits in younger individuals before they progress to more serious problems. To quantify dynamic postural stability during walking, the Margin of Stability (MoS) [10,11] is a widely used indicator due to its strong construct validity. The MoS is grounded in the well-established relationship between the position of the center of mass (CoM) and the base of support (BoS), and it extends this concept to dynamic conditions in which the CoM and postural stability evolve over time. This framework has been applied in numerous studies investigating gait stability and postural control across a wide range of populations and walking situations [12,13,14,15,16,17,18]. In addition to construct validity, MoS also demonstrates criterion-related validity, showing significant associations with fall-related measures [19]. Specifically, MoS correlates with balance assessment tests as well as individuals’ fall history, supporting its utility as a reliable and informative indicator of fall risk. It should be noted that MoS represents dynamic postural stability and is intended to quantify fall risk associated with postural imbalance, rather than to account for all possible causes of falls.

Traditional MoS computation relies on optical motion capture systems, which provide high-resolution kinematic data but are restricted to laboratory environments. In contrast, inertial measurement units (IMUs) have emerged as a practical and scalable alternative for gait and balance analysis [20,21,22,23,24,25,26,27,28,29]. Their portability, low cost, and widespread integration into consumer devices enable continuous monitoring outside controlled settings. Increasing evidence shows that IMU-derived kinematic features can effectively capture stability-related movement characteristics. For instance, Cheng et al. evaluated gait and balance in elderly individuals with knee osteoarthritis using IMUs and demonstrated that absolute angular velocity and linear acceleration were sensitive to postural impairments [20]. Similarly, Lin et al. reported that yoga instructors exhibited superior gait symmetry and single-leg stance stability when assessed with IMUs placed on the trunk and lower limbs [21]. Furthermore, a comprehensive review by Chen et al. highlighted IMU-based sensing as one of the most promising approaches for fall risk assessment due to its flexibility, high temporal resolution, and suitability for long-term monitoring [22].

While IMUs have been widely used to evaluate gait characteristics, only a few studies have attempted to estimate MoS directly from IMU signals. Akiyama et al. [30] demonstrated that MoS categories, that is, high versus low, can be classified using five or six IMUs worn on the body. However, their work addressed neither continuous MoS estimation nor the role of sensor placement in influencing predictive performance. Because MoS reflects whole-body dynamics, identifying optimal IMU locations is essential for capturing relevant movement information. However, existing studies have not clarified how many IMUs are required, nor whether combining signals from multiple body locations provides a clear advantage over a single well-placed sensor. This question is particularly relevant as modern users commonly carry one or two IMU-equipped devices, such as smartphones or smartwatches, in daily environments.

Despite the growing interest in IMU-based gait stability analysis, a systematic understanding of how sensor placement affects MoS estimation remains limited. Prior work based on optical motion capture data has shown that MoS can be accurately estimated from the motion of a single body landmark, with the sacral crest and knee region yielding particularly high accuracy [31]. Nevertheless, it remains unclear whether combining information from multiple body sites can meaningfully improve MoS estimation accuracy beyond that achievable with a single optimally placed IMU. A systematic evaluation of sensor number and placement is therefore lacking.

The present study investigates the influence of sensor placement on MoS estimation by attaching IMUs to ten body regions in young adult participants and using optical motion capture as the reference. By systematically comparing single-sensor and two-sensor configurations, we aim to identify practical sensor placements that maximize predictive accuracy while maintaining minimal sensor usage. Such insight may contribute to the development of wearable stability assessment systems suitable for everyday use and applicable to a wide range of populations.

## 2. Materials and Methods

### 2.1. Gait Data Collection

Participants walked at three speed levels—2.5 km/h, 3.0 km/h, and 3.5 km/h—for approximately three minutes at each speed on a motorized treadmill (FR30z Floatride, Reebok Ltd., Boston, MA, USA). They were instructed to swing their arms naturally and maintain a forward gaze to approximate natural overground walking.

Gait motion was recorded using an optical motion capture system (V120: Trio, NaturalPoint, Inc., Corvallis, OR, USA) at 200 Hz to obtain reference MoS values. Four reflective markers were attached to the participants’ bodies. Two markers were placed on the right and left anterior superior iliac spines (ASIS), and the midpoint of these markers was used as an approximation of the body’s center of mass (CoM), which is close to the whole-body CoM [32,33]. Additionally, a marker was affixed over the region corresponding to the heads of the second and third metatarsals on each foot; these marker positions were used to define the anterior boundary of the base of support (BoS).

Ten IMUs (TSND151, ATR-Promotions Inc., Kansai Science City, Japan) were attached to predefined anatomical locations across the upper and lower body, as described in Section 2.4. Based on our previous findings [34] demonstrating that six-axis signals (three-axis linear acceleration and three-axis angular velocity) provide higher MoS estimation accuracy than three-axis acceleration alone, six-axis IMU data were collected from each sensor at a sampling rate of 100 Hz.

Gait cycles were segmented into right-foot and left-foot stance phases, yielding 60 right-stance steps and 60 left-stance steps per participant for analysis. Steps recorded during the first minute at each speed were excluded to remove transient adaptation effects and ensure steady-state walking data.

### 2.2. Participants

Eight healthy adult males in their twenties participated in the data collection. All participants reported having no injuries or disabilities that could affect their walking ability. Written informed consent was obtained from all participants prior to the experiment.

### 2.3. Ethical Statement

The study protocol was approved by the Institutional Review Board of Tokyo Metropolitan University, Hino Campus (Approval Number: R7-045; Approval Date: 4 September 2025).

### 2.4. Location of IMU Sensors

**Figure 1:**
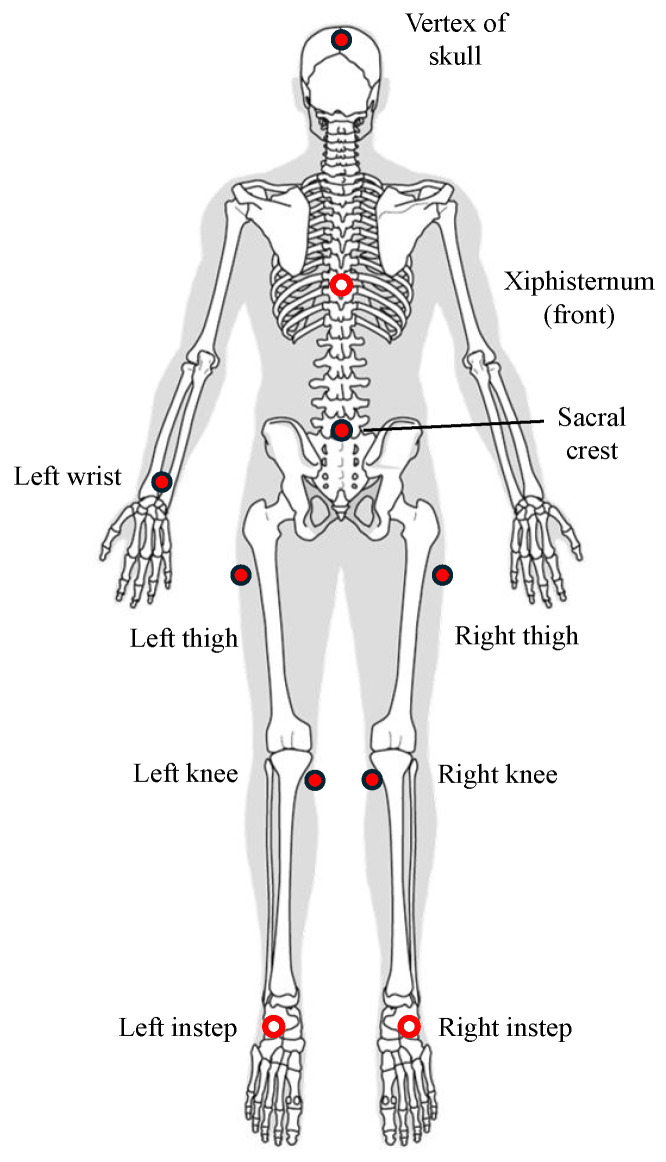
Schematic of the candidate IMU attachment locations examined in this study (frontal view). This figure is intended to provide an overview of the anatomical regions; the exact attachment positions and sensor orientations are shown in Figure 2 and described in Section 2.4. Open circles indicate the feature points on the frontal side. Adapted from [31].

In this study, ten anatomical locations indicated by red markers in Figure 1 were selected for IMU attachment to examine how sensor placement influences gait stability estimation. The selected locations were the skull vertex, xiphisternum, sacral crest, left wrist (dorsal side), bilateral thighs (a point located one third of the thigh length inferior to the greater trochanter), bilateral knees (flat medial surface, slightly distal to the patella), and bilateral insteps.

Among these locations, the skull vertex and left wrist were selected to represent practically feasible sensor positions. The skull vertex enables sensor attachment to a hat or other head-mounted accessory, whereas the left wrist simulates the sensing capabilities of a smartwatch, thereby enhancing real-world applicability.

The sacral crest and bilateral insteps were included because they are located near the CoM and the anterior boundary of the BoS, respectively—two key components used in MoS calculation. In addition, our previous work demonstrated that the knee region provides particularly high estimation accuracy [34]; therefore, bilateral knee sensors were retained as important candidate locations. Sensors on the xiphisternum and bilateral thighs were included as representative upper- and lower-body comparison sites, allowing us to examine how the distribution of body segments influences prediction performance.

Since the present study analyzes gait data separately for left and right stance phases, we aimed to determine whether the laterality of sensor placement affects MoS estimation. Therefore, except for the left wrist—which was intentionally fixed to emulate smartwatch usage—all other sensor locations were instrumented bilaterally, enabling a detailed comparison of left–right effects on MoS prediction.

**Figure 2:**
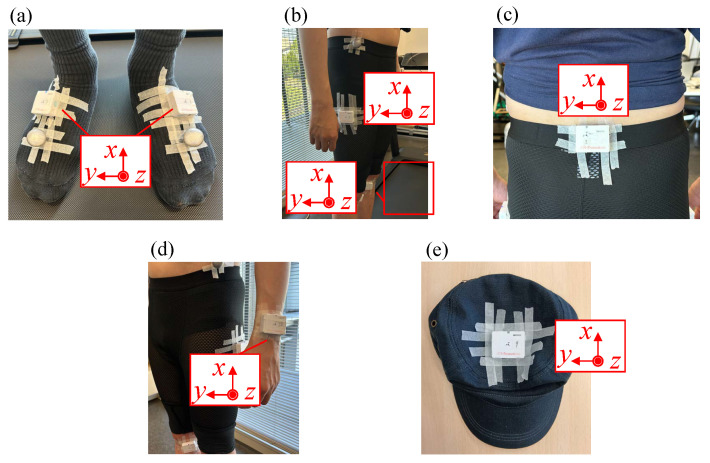
IMU sensors attached to the body parts: (**a**) insteps, (**b**) thighs and knees, (**c**) sacral crest, (**d**) left wrist, and (**e**) skull vertex. Red boxes indicate the coordinate systems of the IMU devices.

Each IMU sensor was firmly affixed to the designated body site, as shown in Figure 2. They were fixed using surgical tape, which is commonly adopted in gait and balance studies to achieve stable attachment while minimizing bulk and relative motion between the sensor and the underlying body segment. All sensors were attached using the same procedure across participants and conditions, thereby reducing systematic bias due to attachment method. No sensor detachment or noticeable displacement was observed during the experiments. Because each sensor had a thin cuboid shape, its largest surface was aligned with a relatively flat portion of the body so that its *z*-axis was oriented normal to the body surface. The *x*-axis of the sensor was then oriented superiorly, except for the sensors attached to the insteps and the skull vertex. The *x*-axes of the sensor placed on the skull vertex and insteps were oriented posteriorly.

### 2.5. Margin of Stability (MoS)

**Figure 3:**
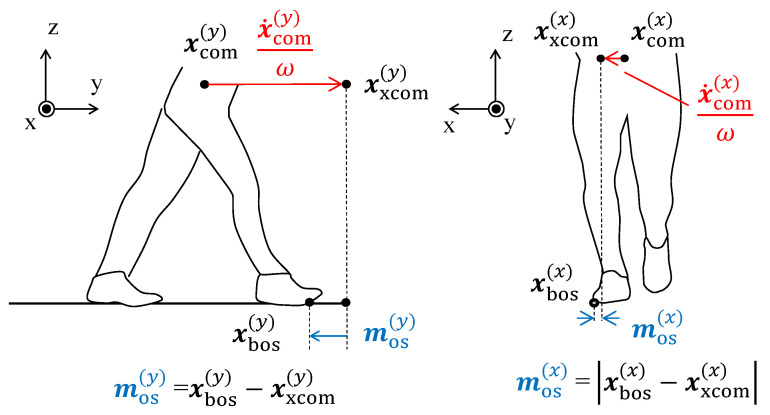
Margin of stability (MoS) on the *x*–*y* plane in the anterior and mediolateral directions. MoS is defined as the vector (anterior) or distance (mediolateral) between the extrapolated center of mass ($x_{\mathrm{xcom}}$) and the boundary of the base of support ($x_{\mathrm{bos}}$). The center of mass is denoted by $x_{\mathrm{com}}$. Red arrows and equations indicate velocity-dependent components. Adapted from [34].

The MoS [10,11] is a widely used metric for assessing dynamic walking stability and evaluating fall risk. It reflects the relationship between the body’s CoM and the BoS, providing insight into the likelihood of losing postural balance. As illustrated in Figure 3, the MoS can be computed in multiple directions [17], including the anterior–posterior and mediolateral directions. This study focuses on both anterior and mediolateral MoS.

The MoS is determined from the position vector of the CoM, denoted by $x_{\mathrm{com}}$, and the position vector of the BoS boundary, denoted by $x_{\mathrm{bos}}$. These vectors are defined on the horizontal (*x*–*y*) plane. The extrapolated Center of Mass (XCoM) on this plane is given by

(1) $$x_{\mathrm{xcom}}=x_{\mathrm{com}}+\frac{{\dot{x}}_{\mathrm{com}}}{\omega },$$

where ${\dot{x}}_{\mathrm{com}}$ is the CoM velocity and ω is the natural frequency of the inverted pendulum model. The natural frequency is computed from gravitational acceleration *g* and the distance from the CoM to the ground *l*:

(2) $$\omega =\sqrt{\frac{g}{l}}.$$

When walking on a treadmill, the belt speed is added to the *y*-component of ${\dot{x}}_{\mathrm{com}}$ [35].

The anterior MoS is defined as the signed difference between the anterior components of the BoS boundary and the XCoM:

(3) $$x_{\mathrm{os}}^{\left(y\right)}=x_{\mathrm{bos}}^{\left(y\right)}-x_{\mathrm{xcom}}^{\left(y\right)}.$$

Here, the superscript (y) denotes the anterior (forward) component of each position vector. A positive value indicates that the XCoM lies within the anterior BoS boundary, corresponding to a stable condition, whereas a negative value indicates that the XCoM has moved beyond the BoS and forward balance is compromised.

In contrast, the mediolateral MoS is defined as the absolute distance between the mediolateral components of the BoS and XCoM:

(4) $$x_{\mathrm{os}}^{\left(x\right)}=\left|x_{\mathrm{bos}}^{\left(x\right)}-x_{\mathrm{xcom}}^{\left(x\right)}\right|.$$

This absolute formulation is used because mediolateral stability is usually above zero and assessed in terms of margin magnitude rather than sign, enabling left- and right-side margins to be treated equivalently. Smaller values reflect reduced mediolateral postural stability.

The MoS evolves continuously throughout the gait cycle, reflecting the moment-to-moment stability of the body. In the literature, researchers commonly analyze either the minimum MoS value within a step, defined as the interval from heel contact of one foot to heel contact of the opposite foot, or the MoS value specifically at heel contact [13,17,18,36,37,38,39,40,41,42]. In the anterior direction, the MoS reaches its minimum at heel contact. In the mediolateral direction, the minimum often occurs at approximately 5–10% (or 55–60%) of the gait cycle, immediately following heel contact [43]. In this study, we adopt the minimum MoS within each step as the target value for analysis.

### 2.6. Principal Motion Analysis to Estimate MoSs

Principal Motion Analysis (PMA) is a supervised learning algorithm used for the analysis of multidimensional time-series data [44], particularly useful for capturing redundant and synergetic movement patterns in tasks such as walking.

**Figure 4:**
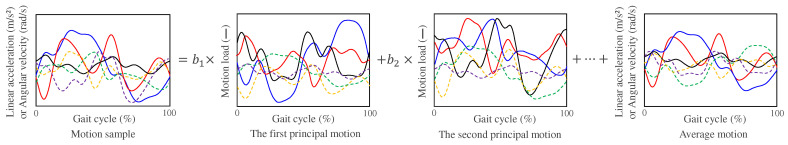
Schematic of the Principal Motion Analysis. A motion sample, consisting of six-axis time-series data, is decomposed into multiple principal motions. The three solid colored curves represent the tri-axial acceleration of a specific bony feature, while the dotted curves represent the corresponding tri-axial angular velocity. $b_{l}$ is the weighting score for the *l*th principal motion.

PMA is a time-series expansion of the Partial Least Squares (PLS) method and identifies common factors or synergies among multiple variables, called principal motions, which represent the fundamental components of the motion data across different individuals or samples. Because PMA inherits the statistical properties of PLS, it is well suited for situations with small-to-moderate sample sizes relative to data dimensionality, which are typical in laboratory-based gait experiments. As shown in Figure 4, these principal motions can be linearly combined to approximate any given walking sample. Each principal motion also presents multidimensional time-series structure. The unsupervised counterpart of PMA, time-series principal component analysis or similar methods, has long been used in gait research to study intersegmental coordination and motor synergies [31,36,45,46,47,48], providing a strong biomechanical foundation for the present approach. The normal gait of healthy individuals can be expressed by a few principal motions (e.g., three) [31,36,45]. Although PMA is a linear method, the principal motions are nonparametric and can assume arbitrarily complex waveform shapes, providing high flexibility in modeling diverse gait patterns.

Because PMA is derived from the PLS framework, it is robust to random measurement noise through dimensionality reduction. By projecting high-dimensional sensor signals onto a small number of principal motions that capture shared variance across variables, uncorrelated noise components are partially averaged out. This property is analogous to the denoising effect observed in time-series principal component approaches commonly used in gait synergy analysis, making PMA well suited for biomechanical signals that contain inevitable sensor noise.

Another practical advantage of PMA is its stability under small-to-moderate sample sizes relative to data dimensionality. Unlike highly parameterized nonlinear models that require large training datasets, PMA restricts model complexity through a low-dimensional representation. In the present study, the number of principal motions was further optimized using cross-validation (see Section 2.7), which provides an additional safeguard against overfitting and improves generalization performance.

From a computational perspective, PMA is lightweight compared with highly parameterized nonlinear learning methods. Model training is performed offline and mainly involves matrix decompositions typical of PLS algorithms. Once trained, MoS estimation requires only a linear projection from the original signal space to the low-dimensional latent space, resulting in computational complexity proportional to the product of input dimensionality and the number of principal motions.

We used 6-axial data in one step as the explanatory values and the minimum anterior and medial–lateral MoS in that step as the objective variable. The time-series data of 6-axial data in a sample (sample *k*) is decomposed into multiple principal motions, each of which is weighted by a score $c_{l,k}$ (l=1,2,…,L, where *L* denotes the number of principal motions used). The principal motions and scores are determined such that the scores and objective variable are correlated by the following procedures.

First, as the explanatory variables, we use the acceleration and angular velocity in or around the *x*, *y*, and *z* axes obtained from *k*th (k=1,…,n) sample as follows:

(5) $$a_{\{x,y,z\},k}={\left[a_{\{x,y,z\},k,0},\ldots ,a_{\{x,y,z\},k,50}\right]}^{⊤}$$

(6) $$\omega_{\{x,y,z\},k}={\left[\omega_{\{x,y,z\},k,0},\ldots ,\omega_{\{x,y,z\},k,50}\right]}^{⊤}.$$

These vectors contain the acceleration and angular velocity at different time points throughout a step sample (0–50%). One step sample is discretized into 51 time points; hence, the size of each vector is 51 ($a_{x,y,z}\in R^{51\times 1},\;\omega_{x,y,z}\in R^{51\times 1}$). We analyzed the right and left steps separately. For each step, the heel contact of the stance leg was defined as 0%, and the heel contact of the opposite (swing) leg was defined as 50%.

Using the velocities in these three directions, we construct the following explanatory variable vector:

(7) $$x_{k}={\left[a_{x,k}^{⊤},\;a_{y,k}^{⊤},\;a_{z,k}^{⊤},\;\omega_{x,k}^{⊤},\;\omega_{y,k}^{⊤},\;\omega_{z,k}^{⊤}\right]}^{⊤}.$$

The size of this extended vector is 306 ($x_{k}\in R^{306\times 1}$).

By combining the 6-axial data vectors from *n* samples for all participants, we formed the explanatory variable matrix $X\in R^{n\times 306}$:

(8) $$X=\left[\begin{array}{c} x_{1}^{⊤}\\ \vdots \\ x_{n}^{⊤} \end{array}\right].$$

By aligning the minimum MoS values for all samples in a column vector, we formed the objective variable vector of size *n* as follows:

(9) $$y={\left[y_{1},\;\ldots ,\;y_{n}\right]}^{⊤}$$

where $y_{k}$ is the minimum MoS value in the *k*th sample.

After centering each column of X and y, they can be approximated as a linear combination of *L* principal motions:

(10) $$X=\sum_{l=1}^{L}c_{l}p_{l}^{⊤}+E$$

(11) $$y=\sum_{l=1}^{L}b_{l}c_{l}+e.$$

Here, $c_{l}={\left(c_{l,1},\ldots ,c_{l,n}\right)}^{⊤}\in R^{n\times 1}$ represents the scores for the *l*th (l=1,…,L) principal motion, $p_{l}\in R^{306\times 1}$ denotes the loadings of the *l*th principal motion, and $b_{l}$ is the regression coefficient corresponding to $c_{l}$. Let E and e be the matrix and vectors of residuals, respectively.

For l≥2, $X_{l}$ and $y_{l}$ are

(12) $$X_{l}=X_{l-1}-c_{l-1}p_{l-1}^{⊤}$$

(13) $$y_{l}=y_{l-1}-b_{l-1}c_{l-1}.$$

Notably, $X_{1}=X$ and $y_{1}=y$.

Here, $c_{l}$, $p_{l}$ and $b_{l}$ are computed as

(14) $$c_{l}=X\frac{X_{l}^{⊤}y_{l}}{\left|X_{l}^{⊤}y_{l}\right|}$$

(15) $$p_{l}=X_{l}^{⊤}\frac{c_{l}}{c_{l}^{⊤}c_{l}}$$

(16) $$b_{l}=\frac{c_{l}^{⊤}}{c_{l}^{⊤}c_{l}}y_{l}.$$

|•| presents the $L_{2}$ norm.

Using the model established by these computations, the MoS of sample *i*, which is not included in the training dataset, can be estimated as follows:

(17) $${\hat{y}}_{i}=x_{i}^{⊤}\sum_{l=1}^{L}b_{l}\frac{X_{l}^{⊤}y_{l}}{|X_{l}^{⊤}y_{l}|}+\bar{y}$$

where $x_{i}$ is the centered explanatory vector for sample *i* and $\bar{y}$ is the mean of the objective variable in the training data set.

The above method assumes the use of 6-axis velocity data from a single IMU as predictors. When using two IMUs, the predictor vector $x_{k}\in R^{612\times 1}$ is extended as follows:

(18) $$x_{k}={\left[a_{1,x,k}^{⊤},a_{1,y,k}^{⊤},a_{1,z,k}^{⊤},\omega_{1,x,k}^{⊤},\omega_{1,y,k}^{⊤},\omega_{1,z,k}^{⊤},a_{2,x,k}^{⊤},a_{2,y,k}^{⊤},a_{2,z,k}^{⊤},\omega_{2,x,k}^{⊤},\omega_{2,y,k}^{⊤},\omega_{2,z,k}^{⊤}\right]}^{⊤}$$

where $a_{1}$, $\omega_{1}$, $a_{2}$, and $\omega_{2}$ denote the acceleration and angular velocity vectors in two different body features (Points 1 and 2), respectively. Using this extended vector, the data matrix X is constructed.

### 2.7. Cross-Validation

To evaluate predictive performance and tune model parameters, we employed 10-fold cross-validation. For each participant, gait samples recorded at the same speed level were evenly distributed across the ten folds. In each fold, one subset was used for testing, and the remaining nine subsets were used for training. The entire 10-fold procedure was repeated five times with different random assignments of samples to folds. Prediction performance was then quantified by averaging the results across all folds and repetitions.

Prediction accuracy for each IMU body location combination was assessed using the root mean squared error (RMSE) between estimated and reference MoS values. The number of principal motions, denoted by *L*, was the unique hyperparameter to be tuned. It was optimized over values from L=1 to L=10 using 10-fold cross-validation. The value yielding the smallest mean prediction error was selected [49], resulting in an optimal value of L=6.

## 3. Results

Table 1 presents the mean and standard deviation of MoS values obtained from optical motion capture in the anterior and mediolateral directions across different walking speeds.

**Figure 5:**
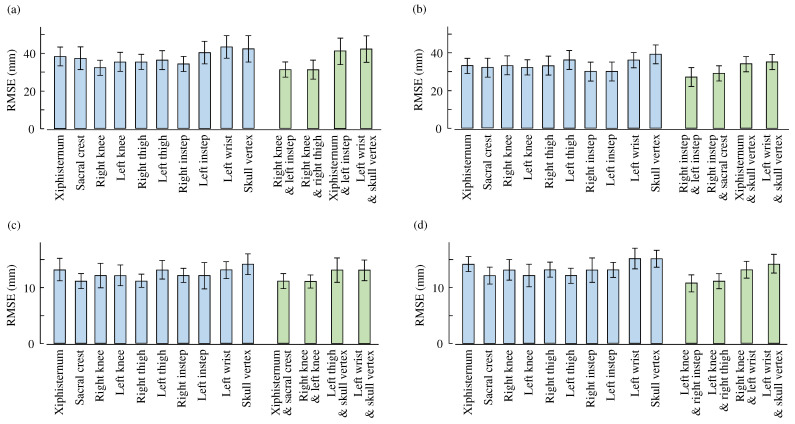
RMSE (mean ± standard deviation) values of different locations. (**a**,**b**) Anterior MoS. (**c**,**d**) Mediolateral MoS. (**a**,**c**) Left-supported steps. (**b**,**d**) Right-supported steps. Blue and green bars are single-IMU and selected dual-IMU conditions, respectively.

Table 2 summarizes the mean and standard deviation of the root mean squared error (RMSE) between the estimated and reference MoS values, together with the mean and standard deviation of the correlation coefficients between the estimated and reference MoS values (shown in parentheses). Diagonal elements represent results obtained using a single body location, whereas off-diagonal elements correspond to combinations of two body locations. For each cell, the first row shows the results when the left foot was the supporting foot, and the second row shows the results when the right foot was the supporting foot. Figure 5 provides a graphical summary of Table 2, highlighting the RMSE values for all single-IMU conditions and selected dual-IMU conditions. For the dual-IMU cases, two representative combinations with the smallest and largest RMSE values are shown.

In the anterior direction, when the left foot served as the supporting foot, the single-location analysis showed that the right knee achieved the lowest RMSE among all body locations, with a value of 32±4mm. Among the two-location combinations, the combination of the right knee and left instep provided the best performance, yielding an RMSE of 31±4mm. Further, the combination of the right knee and right thigh exhibited an RMSE of 31±5 mm. When the right foot served as the supporting foot, the left instep exhibited the best performance among single locations, with an RMSE of 30±5mm. For the two-location combinations under this condition, the combination of the left instep and right instep resulted in the lowest RMSE, reaching 27±5mm.

In the mediolateral direction, when the left foot was the supporting foot, the sacral crest and right thigh showed the best performance among single body locations, achieving an RMSE of 11±1mm. For the two-location combinations, several combinations involving lower-limb segments exhibited comparable estimation accuracy, with RMSE values remaining at 11±1mm. When the right foot served as the supporting foot, the left knee, left thigh, and sacral crest demonstrated relatively good performance among the single-location conditions, with RMSE values of 12±1mm. For the two-location combinations, combinations including the left knee or sacral crest together with selected body locations yielded improved performance, with RMSE values reaching 11±1mm.

## 4. Discussion

First, we discuss whether the estimation accuracy obtained in this study is sufficient for practical MoS analysis. It should be noted that these results are based on data obtained from healthy young adults walking on a treadmill, and their applicability to overground walking or older populations requires further validation. The limitations of this study are summarized in the latter part of this section. As shown in Table 1, in the anterior direction, the standard deviation of the reference MoS distribution across gait samples was approximately 30–40 mm, which closely matched the RMSE of the estimated MoS. Similarly, in the mediolateral direction, the standard deviation of the reference MoS was approximately 15 mm, and the RMSE was slightly smaller than this value.

This correspondence indicates that the estimation error is on the same order as the intrinsic variability of MoS during steady walking. From a practical perspective, such accuracy allows MoS values to be interpreted relative to the population distribution. Specifically, MoS values may be descriptively grouped into ranges such as typical (within one standard deviation from the mean), cautionary (approximately two standard deviations below the mean), and potentially high-risk (below two standard deviations), noting that smaller MoS values indicate higher instability. This categorization should be regarded as an illustrative statistical framework rather than a clinically validated classification. The present study does not include outcome-based validation linking these categories to actual fall events. Instead, the scheme is intended to demonstrate how estimation precision relates to the natural variability of MoS. Establishing clinically meaningful thresholds will require longitudinal studies involving fall incidence and patient populations. Therefore, although the proposed method does not aim to provide highly accurate point-wise MoS values for individual samples, it offers sufficient precision to support distribution-based interpretation.

Although the present analysis focused on the group-level distribution of MoS, it is also informative to consider within-participant variability. The relationship between estimation error and within-participant standard deviation differs across individuals. In the mediolateral direction, for one participant, the smallest RMSE (11 mm) was greater than the within-participant variability of MoS, whereas for others, the RMSE fell below the individual variability. In the anterior direction, the smallest RMSE (27 mm) was greater than the within-participant standard deviation for three of the participants. This observation indicates that the effective resolution of the proposed method depends not only on estimation accuracy but also on the intrinsic variability of gait stability in each individual.

Previous studies have reported that, during treadmill walking, MoS tends to decrease in both anterior and mediolateral directions as walking speed increases [50]. For example, a speed change of approximately ±40% from a preferred walking speed has been shown to induce a change of about 280 mm in the mean anterior MoS and approximately 17 mm in the mean mediolateral MoS. In contrast, findings regarding the relationship between walking speed and MoS during overground walking are less consistent [36,37,38,39,40,50,51].

In our experiment, for the anterior direction, a similar tendency was observed; however, for the mediolateral direction, the mean MoS values did not differ across different speed levels. Across the three treadmill speed levels, the mean anterior MoS varied by approximately 55 mm, as in Table 1. Notably, these velocity-dependent changes are on the same order (or greater) as the estimation error quantified by the RMSE. This correspondence suggests that the proposed method possesses sufficient resolution to detect MoS changes induced by moderate variations in walking speed.

We investigated suitable IMU positions on the body. The head (skull vertex) and wrist locations were found to be less suitable for MoS estimation. One possible reason is that kinematic signals measured at these locations are strongly influenced by individual walking styles, such as arm swing amplitude, arm coordination patterns, and head orientation or motion, which hardly influence the gait stability index.

In contrast, IMUs attached to lower-body segments demonstrated superior performance in MoS estimation. Kinematic information from the thighs, knees, and insteps more directly reflects the mechanical actions involved in gait, such as step placement, support transitions, and lower-limb coordination. These factors are closely linked to the evolution of the base of support and the control of the center of mass during walking. Consequently, signals from lower-body segments are better aligned with the biomechanical mechanisms underlying MoS, leading to more reliable and consistent estimation results.

Based on these findings, the recommended IMU placement depends on the number of available sensors. When only a single IMU is used, the sacral crest represents a reasonable and practical choice. In this case, selecting a location along the body midline is preferable, as unilateral placement on the left or right side may introduce asymmetry-related variability. As a midline location, the sacral crest provides more stable kinematic information than other candidates such as the xiphisternum or the head, while also being relatively easy for users to attach by themselves.

When two IMUs are available, placing sensors on bilateral lower-limb segments—such as the knees, thighs, or insteps—is recommended. IMUs attached to the lower limbs tend to capture gait-related dynamics more directly, resulting in higher estimation accuracy. Across both anterior and mediolateral directions, these bilateral lower-body combinations consistently yielded smaller estimation errors or near-minimal RMSE values. From a practical perspective, such configurations offer a favorable balance between accuracy, robustness against upper-body movement variability, and feasibility for real-world wearable gait stability assessment. Further, the practical applicability of the optimal sensor locations should also be interpreted in the context of real-world walking conditions. While instep-mounted sensors yielded high estimation accuracy under controlled laboratory conditions, real-world overground walking often involves footwear and uneven terrain. In many wearable systems, foot-mounted IMUs are attached near the heel for mechanical stability and user comfort. Therefore, the present results should be regarded as identifying informative locations under ideal attachment conditions, whereas practical deployment may require alternative mounting strategies that balance accuracy and robustness. Validation under ecologically realistic walking conditions remains an important direction for future work.

An important finding of this study was that increasing the number of IMU sensors did not substantially improve the best achievable estimation accuracy, particularly when an optimally placed single sensor was used. In the mediolateral direction, the improvement achieved by adding a second sensor was marginal, on the order of at most approximately 1 mm. These results indicate that sensor placement is far more critical than sensor quantity and suggest that, owing to strong intersegmental synergies, a single IMU can compactly capture whole-body gait dynamics. Such synergies are known to be especially pronounced during cyclic movements such as walking [44,45,47,52,53,54].

Nevertheless, for the anterior MoS, modest but consistent reductions in RMSE were observed when using two sensors compared to a single sensor. Moreover, employing bilateral sensors on the lower limbs may offer practical advantages beyond point-wise accuracy, such as improved robustness across left and right stance phases and greater tolerance to sensor noise. Therefore, while a single optimally placed IMU is sufficient to achieve high estimation accuracy, using two IMUs may provide additional benefits in terms of robustness and bilateral symmetry, although such advantages are not strictly required for accurate MoS estimation.

The present results are comparable to our previous study [31], in which gait data from 60 participants recorded using an optical motion capture system were analyzed. In that study, MoS was predicted from time-series data of three-axial translational velocities obtained from ten bony landmarks. It was reported that using velocity signals from either the knee or the sacral crest yielded the highest estimation accuracy for both anterior and mediolateral MoS. Notably, that study did not investigate combinations of multiple feature points. Although the exact set of measured body landmarks differ between the two studies, both consistently indicate that motion information from the knee and sacral crest is particularly informative for MoS estimation. Despite differences in participant populations, measurement modalities, and experimental environments, the agreement between the two studies supports the generality of these findings.

On the other hand, differences were observed regarding foot-related features. In the previous study [31], velocity information from the toe region (between the second and third metatarsal heads) was found to be relatively less effective, whereas in the present study, velocity signals from the instep provided useful information for MoS estimation. This discrepancy suggests that, for foot-related features, even small differences in measurement location may substantially affect estimation performance.

Several limitations of the present study should be acknowledged.

First, the participants were limited to a small group of young, healthy adult males. Because gait stability characteristics and MoS behavior are known to vary with age [43,55], sex, and clinical condition [56,57], the present findings cannot be directly generalized to older adults or individuals with balance disorders. Accordingly, the present results should be interpreted as a methodological validation of IMU-based MoS estimation and sensor placement effects under controlled conditions, rather than as evidence for direct fall risk classification in clinical populations. Extending the proposed approach to elderly individuals and patient groups will require dedicated validation studies and is an important direction for future work.

Second, all experiments were conducted during treadmill walking. Although treadmill protocols provide controlled and repeatable conditions, the MoS has been reported to differ between treadmill and overground walking [50,58]. In contrast, mediolateral MoS has been reported to be relatively insensitive to differences between treadmill and overground conditions [59]. In general, gait properties differ between the overground and treadmill walking conditions [60]. The present results may not fully reflect gait stability characteristics under natural overground walking.

Third, the CoM was approximated using the midpoint of bilateral ASIS markers due to the simplified motion capture configuration. While this approximation has been adopted in previous studies and is acceptable for relative MoS analysis, it does not represent the exact whole-body CoM, which may affect absolute MoS values.

The present analysis employed a modeling framework based on PMA. Other statistical or machine learning approaches may provide improved estimation performance. Exploring alternative modeling strategies, including more flexible learning frameworks, represents an important direction for future research.

Finally, several considerations should be noted when translating the present findings to real-world smartphone-based applications. First, in practical smartphone-based implementations, the device must be firmly attached to the body, and the relatively large mass of smartphones may induce local skin deformation and additional motion artifacts, potentially affecting signal quality. Second, the experiments were conducted during treadmill walking at constant speeds; however, everyday environments may not provide sufficiently long straight walking paths or stable speed conditions, which could influence the reliability of MoS estimation in uncontrolled settings. Third, the quality and specifications of IMUs embedded in consumer devices vary substantially across manufacturers and models. How differences in sensor noise, bias stability, and sampling characteristics affect MoS estimation accuracy remains unclear. Addressing these issues will require future validation studies using actual consumer-grade devices under naturalistic walking conditions.

## 5. Conclusions

This study examined how IMU sensor placement affects the estimation of the MoS during walking. MoS estimated from six-axis IMU signals using principal motion analysis achieved accuracy comparable to the intrinsic variability of MoS, supporting its use for relative risk assessment. A key contribution of this study is that it systematically quantified the effect of sensor placement on MoS estimation accuracy, identifying sensor configurations that had not been comparatively evaluated before.

Sensor placement had a clear impact on performance. Lower-limb sensors provided the highest accuracy, whereas head and wrist locations were less suitable. The sacral crest is a practical single-sensor option, and bilateral lower-limb placements yield the best two-sensor performance. These findings offer practical guidance for designing wearable gait stability assessment systems and motivate future validation in overground walking and clinical populations.

## Figures and Tables

**Table 1:** Mean and standard deviation of referential MoS values (in millimeters) in the anterior and mediolateral directions.

Speed Level	2.5 km/h	3.0 km/h	3.5 km/h
Anteriro MoS	40.4±42.2	15.8±31.0	−15.4±29.2
Mediolateral MoS	76.8±13.8	77.8±16.0	79.6±16.6

**Table 2:** Mean and standard deviation of the RMSE between estimated and reference MoS values, with correlation coefficients shown in parentheses. Diagonal elements correspond to single-body locations; off-diagonal elements correspond to pairs of locations. For each cell, the first and second rows indicate left- and right-foot support, respectively.

(a) Anterior MoS										
	Xiphisermum	Sacral crest	Right knee	Left knee	Right thigh	Left thigh	Right instep	Left instep	Left wrist	Skull vertex
Xiphisermum	38±5 (0.70±0.06)	35±6 (0.74±0.07)	32±4 (0.79±0.05)	34±4 (0.76±0.07)	33±5 (0.77±0.07)	33±5 (0.75±0.07)	34±5 (0.76±0.07)	41±7 (0.63±0.11)	38±5 (0.69±0.08)	38±5 (0.70±0.08)
	33±4 (0.70±0.06)	31±4 (0.75±0.06)	31±5 (0.74±0.08)	31±5 (0.73±0.08)	31±5 (0.75±0.07)	30±4 (0.76±0.06)	30±4 (0.76±0.05)	30±4 (0.76±0.06)	32±4 (0.72±0.08)	34±4 (0.68±0.06)
Sacral crest		37±6 (0.72±0.08)	32±5 (0.79±0.06)	34±5 (0.76±0.06)	34±6 (0.77±0.08)	35±4 (0.75±0.06)	34±4 (0.77±0.04)	37±7 (0.72±0.10)	39±6 (0.68±0.09)	37±6 (0.71±0.08)
		32±5 (0.72±0.07)	30±5 (0.76±0.07)	30±5 (0.76±0.07)	29±4 (0.77±0.06)	31±5 (0.75±0.08)	29±4 (0.77±0.07)	30±5 (0.77±0.06)	31±4 (0.74±0.06)	32±4 (0.72±0.07)
Right knee			32±4 (0.79±0.05)	32±5 (0.79±0.06)	31±5 (0.81±0.06)	32±4 (0.80±0.06)	33±5 (0.78±0.07)	31±4 (0.81±0.06)	32±5 (0.79±0.06)	33±4 (0.78±0.06)
			33±5 (0.70±0.10)	31±5 (0.74±0.07)	32±5 (0.72±0.08)	31±4 (0.75±0.07)	30±4 (0.75±0.07)	30±5 (0.75±0.08)	32±5 (0.71±0.09)	33±5 (0.70±0.09)
Left knee				35±5 (0.74±0.06)	33±5 (0.78±0.08)	35±4 (0.75±0.06)	33±5 (0.77±0.06)	34±4 (0.76±0.05)	36±5 (0.72±0.08)	36±5 (0.73±0.07)
				32±4 (0.72±0.07)	31±4 (0.74±0.07)	30±4 (0.76±0.07)	28±4 (0.79±0.06)	29±4 (0.77±0.06)	31±5 (0.74±0.09)	32±4 (0.72±0.07)
Right thigh					35±4 (0.75±0.06)	34±5 (0.76±0.08)	32±5 (0.79±0.05)	33±6 (0.78±0.09)	36±6 (0.73±0.07)	35±6 (0.74±0.07)
					33±5 (0.71±0.09)	30±5 (0.75±0.08)	29±4 (0.77±0.05)	30±6 (0.75±0.08)	33±5 (0.69±0.08)	32±5 (0.71±0.08)
Left thigh						36±5 (0.72±0.08)	33±5 (0.77±0.07)	35±6 (0.75±0.08)	37±5 (0.71±0.08)	37±6 (0.71±0.08)
						32±4 (0.72±0.07)	29±4 (0.78±0.06)	29±5 (0.77±0.07)	31±4 (0.75±0.06)	32±5 (0.72±0.08)
Right instep							34±4 (0.76±0.06)	34±4 (0.77±0.06)	33±4 (0.77±0.05)	34±4 (0.76±0.06)
							30±5 (0.75±0.09)	27±5 (0.82±0.06)	30±5 (0.77±0.07)	30±4 (0.75±0.06)
Left instep								40±6 (0.65±0.10)	38±5 (0.69±0.09)	40±6 (0.66±0.10)
								30±5 (0.75±0.08)	30±4 (0.76±0.06)	30±5 (0.75±0.07)
Left wrist									43±6 (0.59±0.12)	42±7 (0.59±0.12)
									36±4 (0.63±0.10)	35±4 (0.65±0.10)
Skull vertex										42±7 (0.60±0.12)
										39±5 (0.54±0.11)
(b) Mediolateral MoS										
	Xiphisermum	Sacral crest	Right knee	Left knee	Right thigh	Left thigh	Right instep	Left instep	Left wrist	Skull vertex
Xiphisermum	13±2 (0.55±0.08)	11±1 (0.70±0.07)	12±2 (0.67±0.11)	12±2 (0.69±0.07)	11±1 (0.69±0.08)	12±1 (0.63±0.10)	12±1 (0.63±0.10)	12±2 (0.66±0.10)	13±2 (0.60±0.11)	13±1 (0.58±0.09)
	14±1 (0.59±0.09)	12±1 (0.70±0.07)	13±2 (0.65±0.10)	12±1 (0.72±0.07)	13±1 (0.67±0.09)	12±2 (0.71±0.08)	13±2 (0.63±0.09)	13±2 (0.67±0.07)	14±1 (0.58±0.09)	13±2 (0.63±0.08)
Sacral crest		11±1 (0.69±0.06)	11±1 (0.71±0.08)	11±2 (0.71±0.08)	11±2 (0.72±0.08)	11±1 (0.70±0.07)	12±1 (0.65±0.06)	12±2 (0.68±0.09)	12±2 (0.67±0.08)	11±2 (0.69±0.07)
		12±1 (0.70±0.05)	12±1 (0.70±0.08)	11±1 (0.75±0.06)	12±1 (0.71±0.07)	11±1 (0.73±0.07)	12±2 (0.68±0.09)	12±1 (0.68±0.07)	12±1 (0.69±0.07)	12±1 (0.72±0.07)
Right knee			12±2 (0.68±0.10)	11±1 (0.72±0.07)	11±1 (0.71±0.09)	12±2 (0.68±0.10)	12±2 (0.67±0.09)	11±1 (0.70±0.09)	12±1 (0.68±0.08)	12±1 (0.68±0.09)
			13±2 (0.61±0.10)	12±1 (0.73±0.07)	13±1 (0.66±0.09)	12±1 (0.71±0.07)	13±2 (0.66±0.10)	12±2 (0.69±0.09)	13±2 (0.65±0.08)	13±2 (0.65±0.08)
Left knee				12±2 (0.66±0.08)	11±1 (0.72±0.09)	12±1 (0.68±0.09)	12±2 (0.66±0.08)	11±1 (0.71±0.08)	12±2 (0.68±0.09)	12±1 (0.66±0.08)
				12±1 (0.72±0.07)	11±1 (0.74±0.06)	11±1 (0.73±0.06)	11±1 (0.73±0.07)	12±1 (0.71±0.09)	11±1 (0.73±0.07)	12±1 (0.73±0.05)
Right thigh					11±1 (0.69±0.08)	11±1 (0.70±0.08)	12±1 (0.66±0.09)	11±1 (0.72±0.07)	12±1 (0.69±0.08)	11±2 (0.70±0.08)
					13±1 (0.64±0.08)	11±2 (0.73±0.07)	12±2 (0.68±0.01)	12±1 (0.71±0.07)	13±1 (0.64±0.09)	13±1 (0.66±0.08)
Left thigh						13±2 (0.61±0.09)	12±1 (0.64±0.09)	12±1 (0.68±0.08)	12±1 (0.64±0.08)	13±2 (0.61±0.10)
						12±1 (0.69±0.08)	12±1 (0.71±0.06)	12±1 (0.71±0.07)	12±1 (0.70±0.06)	12±1 (0.70±0.07)
Right instep							12±1 (0.63±0.07)	12±1 (0.66±0.08)	12±1 (0.65±0.08)	12±1 (0.63±0.10)
							13±2 (0.62±0.09)	12±1 (0.69±0.09)	13±1 (0.63±0.11)	13±2 (0.62±0.11)
Left instep								12±2 (0.62±0.09)	12±1 (0.66±0.08)	12±1 (0.63±0.10)
								13±1 (0.65±0.10)	12±1 (0.69±0.08)	13±2 (0.66±0.11)
Left wrist									13±1 (0.56±0.11)	13±2 (0.58±0.09)
									15±2 (0.48±0.14)	14±2 (0.50±0.14)
Skull vertex										14±2 (0.47±0.10)
										15±1 (0.48±0.11)

## Data Availability

The research data are available from the corresponding author upon direct request, accompanied by a clear explanation of the intended purpose.

## Abbreviations

The following abbreviations are used in this manuscript:

IMU	Inertial Measurement Unit
CoM	Center of Mass
MoS	Margin of Stability
PMA	Principal Motion Analysis
